# Assessing Communicative Effectiveness of Public Health Information in Chinese: Developing Automatic Decision Aids for International Health Professionals

**DOI:** 10.3390/ijerph181910329

**Published:** 2021-09-30

**Authors:** Meng Ji, Adams Bodomo, Wenxiu Xie, Riliu Huang

**Affiliations:** 1 School of Languages and Cultures, The University of Sydney, Sydney 2006, Australia; rhua5035@uni.sydney.edu.au; 2 Department of African Studies, The University of Vienna, A-1090 Vienna, Austria; adams.bodomo@univie.ac.at; 3 Department of Computer Science, City University of Hong Kong, Hong Kong 518057, China; Vasiliky@outlook.com

**Keywords:** health translation, Chinese health resources, readability, health translation, Chinese health resources, readability, accessibility

## Abstract

Effective multilingual communication of authoritative health information plays an important role in helping to reduce health disparities and inequalities in developed and developing countries. Health information communication from the World Health Organization is governed by key principles including health information relevance, credibility, understandability, actionability, accessibility. Multilingual health information developed under these principles provide valuable benchmarks to assess the quality of health resources developed by local health authorities. In this paper, we developed machine learning classifiers for health professionals with or without Chinese proficiency to assess public-oriented health information in Chinese based on the definition of effective health communication by the WHO. We compared our optimized classifier (SVM__F5_) with the state-of-art Chinese readability classifier (Chinese Readability Index Explorer CRIE 3.0), and classifiers adapted from established English readability formula, Gunning Fog Index, Automated Readability Index. Our optimized classifier achieved statistically significant higher area under the receiver operator curve (AUC of ROC), accuracy, sensitivity, and specificity than those of SVM using CRIE 3.0 features and SVM using linguistic features of Gunning Fog Index and Automated Readability Index (ARI). The statistically improved performance of our optimized classifier compared to that of SVM classifiers adapted from popular readability formula suggests that evaluation of health communication effectiveness as defined by the principles of the WHO is more complex than information readability assessment. Our SVM classifier validated on health information covering diverse topics (environmental health, infectious diseases, pregnancy, maternity care, non-communicable diseases, tobacco control) can aid effectively in the automatic assessment of original, translated Chinese public health information of whether they satisfy or not the current international standard of effective health communication as set by the WHO.

## 1. Introduction 

Effective multilingual communication of authoritative health information plays an important role in helping to reduce health disparities, inequalities in developed and developing countries. Currently, the evaluation of the quality of public-oriented health information focused on readability assessment [1,2,3,4,5,6,7]. In 2013, the Agency for Healthcare Research and Quality of the U.S. Department of Health and Human Services developed the Patient Education Materials Assessment Tool (PEMAT) which extended the evaluation of the quality of user-oriented health information from readability, understandability to include actionability [8,9]. PEMAT provided detailed guidelines for qualitative patient-oriented health information understandability analysis: word choice and language style (use of common, familiar terms, active voice), numeracy (use of easy-to-understand numbers and measurements; no calculations), information organization (break chunks of information to short sections; logical sequence of information; use of informative headers and summary), layout, design and use of visual aids. Actionability is assessed through whether the materials contain clearly defined actions, break actions into manageable, explicit steps, use of visual or graphical aids. 

More recently, The World Health Organization developed The Strategic Framework for Effective Communications [10,11,12]. The Framework significantly enriched the dimensions of the evaluation of the quality of online health contents intended for health decision makers at all levels including individuals and communities from diverse language, cultural, educational, and socio-economic backgrounds. In this Framework, six large principles are highlighted for developing effective health resources: information accessibility, understandability, actionability, credibility, relevance, and timeliness. Among these principles, five are directly related to online health information quality assessment: understandability, actionability, credibility, relevance, and timeliness. This represents a further extension of the PEMAT guidelines focusing on the first two criteria: understandability, actionability. 

Within the WHO Strategic Framework, understandability was assessed in terms of use of plain, familiar language; actionability was associated with the use of messages that are simple, easy to recall, repeated and attention getting; messages that can facilitate the understanding of health risks, specify the steps needed to protect people’s health; messages that help people to implement protective measures by describing the desired action and explaining where to find information and resources that support implementation. Information creditability mandates that fact sheets are updated to ensure technical accuracy and that readers can identify whether they are at risk, understand the scale of the problem, learn ways they can protect their health and safety, recognize the barriers to improvement, recognize the barriers to improvement. Transparency is part of information credibility, particularly for scenarios of high uncertainty. To ensure information credibility, public health information needs to clarify what information is known and what is not known; emphasize that interim public health recommendations could change as new information becomes available, and let audiences know about the limitations of the conclusions from preliminary research findings. Information relevance requires the tailoring of health messages to make the content relevant to the specific audience; address the barriers people may face when trying to take recommended action. This is to engage the target audience, communicate risks effectively, and achieve the desired single overarching communications outcome (SOCO). Lastly, timeliness requires that under public health emergencies, quick release of health information should include symptoms, severity of the threat, where the threat is located or spreading, who is at risk, how people can protect themselves, what is being done to contain the threat, and how to get answers for questions. 

## 2. Methods

### 2.1. Research Design 

The general principles and implementation strategies for these principle in the WHO Strategic Framework for Effective Communications lay the foundation for more integrated assessment of online public-oriented health contents. However, without detailed guidelines and quantitative tools, the use of the Framework in practical health information assessment remains a real challenge, especially for languages which lack in standardized readability assessment tools even for basic understandability check. Chinese is one of the two non-European languages (with Arabic) among the six official languages of the WHO (English, Spanish, Russian, French). Despite an increasing interest in quantitative readability assessment of Chinese educational materials, currently there is no internationally validated Chinese readability tools. As a result, the evaluation of the quality of health information in Chinese remains largely understudied nor benchmarked against international health promotion resources in the same language. At the WHO, translations serve as an important channel for the dissemination of public health information. To control for the variability of translated contents, translation professionals at the WHO use enhanced translation strategies (forward and backward translation) to produce multilingual contents of original English health materials [13,14]. In our study, we collected high-quality WHO Chinese translations of health contents intended for the public in sections of the WHO website such as Health Topics, Fact Sheets, Q&As. The WHO Strategic Framework for Effective Communications specifies and highlights that these materials have been developed under the principles of effective health communication of accessibility, understandability, actionability, credibility, relevance, and timeliness.

In our study, we collected and used these materials which have been developed to reflect the principles of effective health communication by WHO health professionals and then thoroughly translated to other official languages including Chinese as training materials to develop machine learning classifiers. The main function of these classifiers was to aid in the automatic evaluation of the communicative effectiveness of health information in Chinese from non-WHO sources, mainly health authorities and health research institutes in China. We trained and validated the classifier on WHO and non-WHO exiting Chinese health materials on a range of diverse health topics including environmental and occupational health, infectious diseases, pregnancy and maternity care, non-communicable disease, tobacco control to ensure its wide applicability. To validate our classifier, we further compared the performance of our classifier with that of the state-of-the-art Chinese readability classifier using Support Vector Machine (SVM, implemented in the Python scikit-learn package, available at https://scikit-learn.org/stable/modules/generated/sklearn.svm.SVC.html) (accessed on 1 June 2021) [15,16] and Chinese Readability Index Explorer (CRIE 3.0), as well as SVM classifiers using features from well-established English readability formula, Gunning Fog Index and Automated Readability Index (ARI), as other English readability tools are not readily adaptable to the Chinese language materials. 

### 2.2. Study Materials Collection 

A comparable corpus was constructed which contained a large number of health texts of WHO and China health information from credible, authoritative Chinese sources such as national and local research centers, and not-for-profit organizations of health promotion in China (Appendix A). These Chinese health resources were however not developed under the principles of Strategic Framework for Effective Communications. Chinese health materials from the WHO were Chinese translations from original English materials. Efforts were made to maintain the structural balance between the two sets of data in terms of topical similarity. Some topics were well represented in both sources of Chinese health information such as infectious diseases, pregnancy and maternity, environmental health, non-communicable diseases, and some topics were absent from Chinese health sources such as mental health, dementia, antibiotics, capacity building, accidents. This was partly due to the global reach of the WHO and the diversity of health topics covered by the international health organization, and partly due to the lack of relevant health educational materials on topics such as mental health, dementia, disability from Chinese health authorities. After screening for health texts which were present in both sources, the total number of health texts collected for the corpus was 578: WHO resources (352) and non-WHO Chinese health sources (226).

### 2.3. Related Work 

Currently, the assessment of the quality of health resources for readers is based on established readability tools and formula including Flesch Reading Ease Score [17], Gunning Fog [18], Flesch-Kincaid Grade Level, Coleman-Liau Index [19], SMOG Index [20,21], Automated Readability Index [22], Linseed Write Formula. The advantages of these tools consist in their convenience and rapid assessment of the suitability of the written materials for people with varying educational levels. These tools have been used extensively in the quantitative evaluation of specialised and general health materials for many decades. However, a major limitation of these tools is that they have not been validated in non-European languages and that some of the linguistic features such as average word length in syllables used in these tools are not readily adjustable to languages using non-alphabetical writing symbols like Chinese. In recent years, with the rapid development of natural language processing techniques, research in the automatic assessment of Chinese written materials has been developing rapidly. The Chinese Readability Index Explorer was used which was developed by National Taiwan Normal University [23,24]. CRIE enables high-dimensional, integrated linguistic analyses of Chinese texts, and ranks their readability (for Chinese readers from Year 1 to 12 educational backgrounds) according to the assessment score of individual linguistic items. 

## 3. Results

### 3.1. Statistics

In our study of developing machine learning classifiers to diagnose and predict the overall communicative effectiveness of Chinese health resources using existing WHO Chinese information as benchmarks, we used the latest version of the Chinese Readability Index Explorer (version 3.0). Table 1 shows the 29 word/lexical, syntactic, semantic and cohesion features of the system. Mann Whitney U test was used to compare the statistical significance of the differences between WHO and non-WHO Chinese public health information. 

The mean of almost 55% (16) of the total linguistic features were statistically different. The means of 11 linguistic features in non-WHO Chinese health resources were significantly higher that of WHO Chinese health resources: average sentences per paragraph (mean difference M = 1.79, *p* < 0.001); difficult words (M = 30.07, *p* < 0.001), middle-stroke characters (M = 19.05, *p*,0.001), average strokes per character (M = 0.04, *p* < 0.001), number of sentences (M = 6.75, *p* < 0.001), number of content words (M = 43.83, *p* < 0.001), sentences with complex semantic categories (M = 5.02, *p* < 0.001), density of content words (M = 0.03, *p* < 0.001), number of 3-character words (M = 2.71, *p* < 0.001), number of words (M = 47.37, *p* = 0.003), and number of characters (M = 71.84, *p* = 0.013). The means of 5 linguistic features in WHO Chinese resources were statistically higher than those of non-WHO Chinese resources: average words per sentences (M = 1.28, *p* < 0.001), number of single sentences (M = 1.28, *p* < 0.001), number of positive conjunctions (M = 1.31, *p* < 0.001), ratio of noun phrases (M = 0.06, *p* < 0.001), number of paragraphs (M = 0.79, *p* < 0.001). The means of the remaining linguistic features in the two sets of Chinese health texts were insignificant with *p* larger than 0.05. These findings suggest that non-WHO original Chinese health resources were more complex orthographically, lexically, and syntactically and information was presented in larger chunks of paragraphs. By contrast, WHO Chinese resources developed under the principles of information accessibility, actionability, understandability and relevance were statistically less complex in terms of use of difficult words, the average number of strokes (similar to English letters) per Chinese character (similar to English words), the use of sentences containing semantically complex expressions such as polysemous words which are likely to cause confusion or conceptual mistakes. The statistically higher means of 3-character words (such as proper nouns, names of diseases, medicines, fixed expressions), and higher ratios of content words in non-WHO original Chinese materials suggests higher cognitive loads of original Chinese public health materials. By contrast, the results demonstrated that WHO Chinese health materials were logically more coherent (use of positive conjunctions). The potential barrier to understand WHO materials was the statistically higher ratios of noun phrases, which could be explained partly by the impact of the original English materials on the Chinese translations, and partly by the specialised genre of medical and health materials. 

Table 2 shows the effect sizes (Hedges’g) and common language effect size (CLES) [25] of calcaulated for linguistic features which had statistically signifincant differnces in two sets of Chinesa health materials following Mann Whitney U test. It shows that despite that the means of 13 linguistic features were statistically significant (*p* < 0.05), the effect sizes (Hedges’ g) of eight features were larger than 0.5 as medium effect sizes. Larger Hedges’ g and CLES were indicators of the discrimination effectiveness of the features to separate between WHO and non-WHO Chinese materials. Comparison of effect sizes helped us to further reduce the number of features to be used in training machine learning classifier, i.e., SVM with linear kernel. 

### 3.2. Classifier Optimisation

#### Stepwise Regression

After the examination of the effect sizes of linguistic features, we continued with feature optimisation using conditional stepwise logistic regression (software SPSS version 26). Table 3 shows the schedule of forward stepwise modelling of 8 linguistic features which had both statistifcally significant *p* values (<0.05) and medium to large effect size (Hedges’ g > 0.5, CLES > 0.6): density of content words, average sentences per paragraph, Sentences with complex semantic categories (polysemeous words), average words per sentences, difficult words, single sentences, middle-stroke characters, average strokes per character. 

The final model selected five features, as they contributed significantly to the model and three linguistic features were eliminated for being statistically insignificant [26]. The sequence of features entering the model was average sentences per paragraph, density of content words, average strokes per character, average words per sentences, single sentences. 

**Figure 1:**
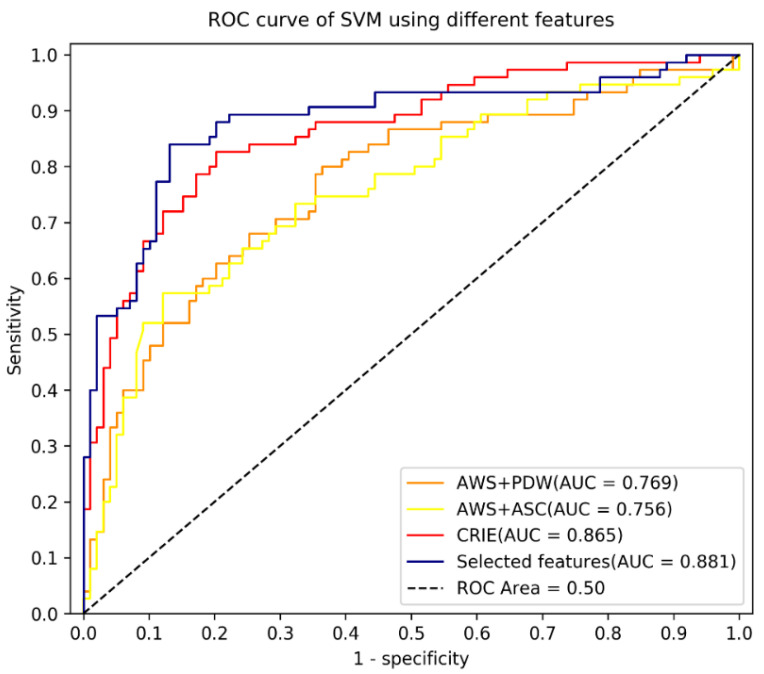
Area under receiver operating characteristic of SVM with different features.

Using statistical significance (two tailed *p* values and effect size Hedges’ g), and forward stepwise regression modelling, we optimized the feature set by reducing the original 29 features (Table 1) of the Chinese Readability Explorer Index (CRIE 3.0) to five linguistic features (Table 3) contributing statistically significantly to the model. We compared the performance of the two SVM classifiers using the original feature set (SVM_29) with the optimized classifier (SVM_5). To further validate our model, we adapted widely used readability formula to machine learning classifiers: SVM_GFI (using linguistic features from the Gunning Fog Readability Index) and SVM_ARI (using linguistic features from the Automated Readability Index ARI). There were two features in SVM_GFI: Average Words per Sentences (AWS) and Average Strokes per Character (ASC); and 2 features in SVM_ARI: Average Words per Sentences (AWS) and percentage of difficult words (PDW). We compared the accuracy, sensitivity, specificity of our model with the 3 referential classifiers on the testing dataset (Table 4), and the area under the receiver operator curve (Figure 1).

Table 5, Table 6 and Table 7 show the results of paired sample t tests of the accuracy, sensitivity and specific of the 4 classifiers on the testing dataset: SVM_5 for the optimize linear kernel SVM, SVM_29 for the unoptimized classifier with the full feature set (29 in total) of CRIE 3.0 system, SVM_GFI adapted from Gunning Fog Readability Index and SVM_ARI adapted from Automated Readability Index (ARI). We adjusted the significance level from 0.05 to 0.0083 using Bonferroni correction to counteract the problem of multiple comparisons, that is, *p* value is statistically significant if smaller than 0.0083 only. It shows that the optimized classifier SVM_5 achieved statistically higher accuracy than the unoptimized SVM_29 using full CRIE features (*p* = 0.0035), SVM_GFI (*p* = 0.0015) and SVM_ARI (*p* = 0.0020); SMV_5 had statistically higher sensitivity than SVM_29 (*p* = 0.0038), SVM_GFI (*p* = 0.0031) and SVM_ARI (*p* = 0.0032); and SVM_5 was statistically improved in specificity when compared to SVM_29 (*p* = 0.0030), SVM_GFI (*p* = 0.0009) and SVM_ARI (*p* = 0.0014). 

## 4. Discussion

The state-of-the-art machine learning classifiers for Chinese written materials is the Chinese Readability Index Explorer (CRIE) which has been developed, tested in the classification of Chinese language educational materials. Recent studies by Sung, et al. [23,24] developed machine learning algorithms to classify text materials for students from Year 1 to Year 12 in the primary and secondary education in Taiwan. The best-performing machine learning classifier developed in Sung, et al. was a support vector machine with radial basis function (RBF) kernel. Using 31 linguistic features from the 75 features of the CRIE 1.0 system, the support vector machine (SVM_31) reported an average accuracy of 71.75% in classifying Chinese language arts textbooks from Year 1 to Year 6 levels. The 31 features included in the SVM model encompassed nine at word level to measure character, lexical complexity, five measuring semantic complexity, six measuring syntactic complexity, and 21 measuring textual cohesion. We replaced RBF with linear kernel to reduce complexity and increase the generalizability and computational efficiency of our SVM model, as the size of our dataset doubled that of the earlier study. We adapted the SVM RBF model to a linear kernel SVM using the refined feature set of the CRIE 3.0 system which had as many as 29 features measuring orthographic complexity (average strokes per character, 2-character words, 3-character words, low-stroke characters, middle-stroke characters, high-stroke characters), lexical complexity (average idioms per sentences, difficult words, sentences with complex semantic categories), information load (single sentences, average sentences per paragraph, average words per sentences), cognitive load (content words, density of content words, average logarithmic frequency of content words, ratio of noun phrases, frequency of noun phrases per 10 K), information cohesion (positive conjunctions, personal pronouns, negative conjunctions, adverbs of negation, conjunctions, pronouns). 

Our optimized SVM classifier contained only five linguistic features to effectively separate WHO Chinese health resources of higher communicative effectiveness as defined by the Strategic Framework of Effective Communication, and non-WHO Chinese health materials for public communication purposes. The five features model highlighted the key dimensions which distinguished Chinese health information of WHO defined communicative effectiveness from those not: orthographic complexity (average strokes per character); information load (single sentences, average sentences per paragraph, average words per sentences), and cognitive load (density of content words). The exclusion of linguistic features which had large effect sizes such as number of difficult words, sentences with complex semantic categories from the optimized SVM model suggests that machine learning differs from statistical modelling, as features which had statistically different distributions in the outcome datasets might not be best discriminating features for machine learning. 

The result of the comparison between our optimized SVM classifier with the unoptimized classifier using the CRIE 3.0 features shows that the performance of our model improved on the testing data in terms of model AUC, overall classification accuracy, sensitivity to detect Chinese health texts not aligning with WHO defined communicative effectiveness, as well as specificity to diagnose Chinese health texts matching the WHO defined communicative effectiveness, thus suitable for the Chinese-speaking readership globally. We could not directly assess the actual readability levels of WHO and non-WHO Chinese health materials due to lack of internationally validated readability formula for Chinese. We adapted English readability formula Gunning Fog Readability Index and Automated Readability Index to linear SVM classifiers to further validate our optimized classification model. The results again attested to the efficiency and high performance of our model, with statistically much higher accuracy, sensitivity, and specificity for clinical use. 

In sum, the result shows that our new classifier achieved statistically higher accuracy, sensitivity, and specificity compared to the state-of-the-art machine learning models for classification of Chinese primary, secondary educational materials, as well as linear kernel classifiers adapted from well-established readability formula: Gunning Fog Readability Index, and Automated Readability Index (ARI). Our classifier provided a needed quantitative tool for assessing and predicting the membership of a certain Chinese health text in terms of its communicative effectiveness, that is, whether it aligns with the current level of communicative effectiveness of existing WHO public-oriented health in-formation in Chinese. 

## 5. Conclusions

Improving the communicative effectiveness of multilingual health resources can help reduce the increasing health inequalities and disparities among multilingual, multicultural communities. Despite that readability has a long history in English health resource quality evaluation, the quantitative evaluation of health resources is understudied in languages using distinct writing systems like Chinese. Furthermore, the complexity of effective health communication based on health research insights has now gone beyond readability and understandability. The WHO Strategic Framework for Effective Communications stipulates new principles such as information accessibility, actionability, relevance, credibility, timeliness. Developing separate quantitative instruments to measure these dimensions could be challenging, and theoretically misleading, as specific implementation strategies and methods may well overlap among these large principles. 

Developing new research instruments like machine learning algorithms can help national and global health professionals to assess the quality of multilingual public health resources in a more integrated, adaptive fashion with significantly improved accuracy, precision, and reliability [27,28], as these are known advantages of machine learning classifiers which are having increasing applications in wide-ranging research fields. To overcome the issue of the difficulty to interpret machine learning models, also to increase the generalizability of our newly developed instrument, we used sparce linear classifiers such as SVM which tends to outperform other highly complex machine learning classifiers with small feature sets. 

We hope to provide international health professionals engaged in multilingual health communication, with or without Chinese proficiency, with much-needed decision aids to assess whether a certain health promotion text in Chinese is suitable for general Chinese-speaking readers, using existing WHO Chinese materials developed under its Strategic Framework for Effective Communications as the international benchmarks of multilingual health communication effectiveness.

The simple, easy-to-interpret nature of our tool can be effectively adapted to other languages, especially languages spoken by communities for whom high-quality health information is urgently needed to help reduce increasing health inequalities caused by persistent lack of quality health information and lack to healthcare services.

## 6. Limitations

We would like to acknowledge the limitations of our study. The definition of the WHO principles of effective health communications is qualitative and currently very general. Important differences, diversity do exist among populations, communities, and individuals in terms of education, native language and English proficiency, health literacy, socio-economic status, cognitive ability, mental and physical health, cultures, and other limiting factors. To improve the adaptability, precision of quantitative and computational tools to assess the suitability of health materials, there is a need to develop guidelines and evaluation criteria which capture the practical, diverse needs from diverse people and communities. With better targeted evaluation guidelines from health authorities and high-quality materials in English and their translations to other languages, we could improve the computational models to better support evidence-based health information assessment.

## Figures and Tables

**Table 1:** Mann Whitney U Test.

Feature	WHO Chinese		Non-WHO Chinese		*p*
	Mean	SD.	Mean	SD.	
Average sentences per paragraph	3.32	1.16	5.11	2.74	*p* < 0.001
Difficult words	70.87	35.99	100.94	65.30	*p* < 0.001
Middle-stroke characters	51.81	27.70	70.86	49.39	*p* < 0.001
Average strokes per character	7.71	0.34	7.87	0.31	*p* < 0.001
sentences	17.20	8.60	23.95	16.67	*p* < 0.001
Average words per sentence	11.89	1.96	10.61	2.02	*p* < 0.001
Single sentences	0.46	0.19	0.35	0.21	*p* < 0.001
Sentences with complex semantic categories	7.61	4.76	12.63	9.74	*p* < 0.001
Density of content words	0.81	0.03	0.84	0.04	*p* < 0.001
Positive conjunctions	9.00	5.19	7.69	5.51	*p* < 0.001
Ratio of noun phrases	0.41	0.20	0.35	0.17	*p* < 0.001
Content words	163.07	80.54	206.90	140.3	*p* < 0.001
3-Character words	8.30	6.37	11.01	8.55	*p* < 0.001
Paragraph numbers	5.57	3.08	4.78	2.12	0.001
Words	200.49	99.76	247.86	170.3	0.003
Characters	337.64	166.0	409.48	283.1	0.013
Low-stroke characters	284.71	140.9	337.45	236.5	0.059
Adverbs of negation	0.93	1.23	1.26	1.77	0.135
2-Character words	114.34	56.26	132.95	96.94	0.221
TTR	0.62	0.08	0.62	0.09	0.264
High-stroke characters	0.45	1.60	0.44	1.31	0.281
Conjunctions	11.52	6.41	11.82	8.44	0.357
Frequency of noun phrases per 10 K	314.66	37.00	315.61	40.08	0.428
Average idioms per sentences	0.01	0.03	0.01	0.03	0.453
Personal pronouns	0.70	1.14	1.04	2.62	0.714
Idioms	0.22	0.51	0.28	0.81	0.720
Negative conjunctions	1.44	1.39	1.57	1.95	0.733
Pronouns	1.47	1.65	1.91	3.25	0.819
Average logarithmic frequency of content words	1.34	0.18	1.34	0.20	0.903

**Table 2:** Computation of Effect Sizes Hedges’g and Common Language Effect Size (CLES).

Features	*p*	Hedges’ g	CLES
Density of content words	0	−1.569	0.866
Average sentences per paragraph	0	−0.927	0.744
Sentences with complex semantic categories	0	−0.703	0.69
Average words per sentence	0	0.646	0.676
Difficult words	0	−0.607	0.666
Single sentences	0	0.555	0.653
Middle-stroke characters	0	−0.506	0.64
Average strokes per character	0	−0.477	0.632
Content words	0	−0.406	0.613
3-Character words	0	−0.371	0.604
Ratio of noun phrases	0	0.335	0.594
Ratio of noun phrases	0	0.335	0.594
Positive conjunctions	0	0.247	0.569

**Table 3:** Stepwise Regression (Variables in the Equation).

Variables in the Equation					
Steps	Features	B	S.E.	Wald	Sig.
Step 1	Average sentences per paragraph	−0.578	0.066	76.713	*p* < 0.001
	Constant	2.754	0.275	99.945	*p* < 0.001
Step 2	Average sentences per paragraph	−0.667	0.074	80.136	*p* < 0.001
	Density of content words	−25.597	3.202	63.926	*p* < 0.001
	Constant	24.269	2.753	77.726	*p* < 0.001
Step 3	Average sentences per paragraph	−0.714	0.077	85.171	*p* < 0.001
	Average strokes per character	−1.873	0.346	29.287	*p* < 0.001
	Density of content words	−24.539	3.279	56.002	*p* < 0.001
	Constant	38.172	4.004	90.896	*p* < 0.001
Step 4	Average sentences per paragraph	−0.635	0.079	64.680	*p* < 0.001
	Average strokes per character	−2.061	0.356	33.506	*p* < 0.001
	average words per sentences	0.254	0.063	16.143	*p* < 0.001
	Density of content words	−23.123	3.342	47.858	*p* < 0.001
	Constant	35.318	4.087	74.659	*p* < 0.001
Step 5	Average sentences per paragraph	−0.595	0.080	55.487	*p* < 0.001
	Average strokes per character	−2.012	0.358	31.633	*p* < 0.001
	average words per sentences	0.218	0.064	11.561	0.001
	Single sentences	1.821	0.566	10.353	0.001
	Density of content words	−24.416	3.412	51.203	*p* < 0.001
	Constant	35.516	4.093	75.279	*p* < 0.001

**Table 4:** Validation performance of SVM with different feature sets on testing data.

Method	Accuracy Mean (SD)	Sensitivity	Specificity
SVM_GFI	0.678 (0.055)	0.748 (0.057)	0.568 (0.052)
SVM_ARI	0.714 (0.054)	0.757 (0.056)	0.648 (0.050)
SVM_29	0.799 (0.048)	0.792 (0.053)	0.810 (0.041)
SVM_5	0.849 (0.043)	0.841 (0.048)	0.861 (0.036)

**Table 5:** Paired sample t test of the accuracy of SVM classifiers.

Pair	Classifiers	Paired Differences			95% C.I. of the Difference		Sig. (2-Tailed)
		Mean	S.D.	S.E.	Lower	Upper	
1	SVM _5 vs. SVM_29	0.0496	0.0051	0.0029	0.0371	0.0622	0.0035
2	SVM _5 vs. SVM_GFI	0.1710	0.0115	0.0066	0.1424	0.1996	0.0015
3	SVM _5 vs. SVM_ARI	0.1345	0.0104	0.0060	0.1087	0.1603	0.0020
4	SVM_29 vs. SVM_GFI	0.1214	0.0065	0.0037	0.1053	0.1374	0.0009
5	SVM_29 vs. SVM_ARI	0.0849	0.0053	0.0031	0.0717	0.0981	0.0013
6	SVM_GFI vs. SVM_ARI	−0.0365	0.0011	0.0007	−0.0393	−0.0337	0.0003

**Table 6:** Paired sample *t* test of the sensitivity of SVM classifiers.

Pair	Classifiers	Paired Differences			95% C.I. of the Difference		Sig. (2-Tailed)
		Mean	SD	S.E.	Lower	Upper	
1	SVM _5 vs. SVM_29	0.0487	0.0052	0.0030	0.0357	0.0616	0.0038
2	SVM _5 vs. SVM_GFI	0.0929	0.0089	0.0051	0.0708	0.1151	0.0031
3	SVM _5 vs. SVM_ARI	0.0841	0.0082	0.0048	0.0636	0.1045	0.0032
4	SVM_29 vs. SVM_GFI	0.0442	0.0037	0.0021	0.0350	0.0535	0.0023
5	SVM_29 vs. SVM_ARI	0.0354	0.0030	0.0017	0.0279	0.0429	0.0024
6	SVM_GFI vs. SVM_ARI	−0.0088	0.0007	0.0004	−0.0105	−0.0072	0.0019

**Table 7:** Paired sample *t* test of the specificity of SVM classifiers.

Pair	Classifiers	Paired Differences			95% C.I. of the Difference		Sig. (2-Tailed)
		Mean	SD	S.E.	Lower	Upper	
1	SVM _5 vs. SVM_29	0.0511	0.0048	0.0028	0.0391	0.0632	0.0030
2	SVM _5 vs. SVM_GFI	0.2926	0.0156	0.0090	0.2539	0.3313	0.0009
3	SVM _5 vs. SVM_ARI	0.2131	0.0137	0.0079	0.1789	0.2472	0.0014
4	SVM_29 vs. SVM_GFI	0.2415	0.0107	0.0062	0.2148	0.2681	0.0007
5	SVM_29 vs. SVM_ARI	0.1619	0.0089	0.0051	0.1398	0.1840	0.0010
6	SVM_GFI vs. SVM_ARI	−0.0795	0.0018	0.0011	−0.0841	−0.0750	0.0002

## Data Availability

Not applicable.

## Appendix A

Infectious Disease Control and Prevention Institute

Viral Disease Control and Prevention Institute

Parasitic Disease Prevention and Control Centre

STD and AIDS Prevention and Control Centre

Chronic Non-communicable Disease Prevention and Control Centre

Nutrition and Health Institute Environment and Health Related Product Safety Institute

Institute of Occupational Health and Poison Control Radiation Protection and Nuclear Safety Medicine

Rural Water Improvement Technology Guidance Centre

Maternal and Child Health Centre)

China Association for STD and AIDS Prevention and Control

Chinese Nutrition Society

China Hepatitis Prevention Foundation

Preventive Medicine Information Committee of Chinese Preventive Medicine Association
